# Trends in Cardiac Pacemaker Batteries

**Published:** 2004-10-01

**Authors:** Venkateswara Sarma Mallela, V Ilankumaran, N.Srinivasa Rao

**Affiliations:** * Dept. of Electronic and Computer Engineering, Caledonian College of Engineering, (affiliated to Glasgow Caledonian University, UK), Sultanate of Oman; † Dept. of Electronics and Communications Engineering, Bhoj Reddy Engineering College for Women,(affiliate to Jawaharlal Nehru Technological Universisy), Hyderabad, India

## Abstract

Batteries used in Implantable cardiac pacemakers-present unique challenges to their developers and manufacturers in terms of high levels of safety and reliability. In addition, the batteries must have longevity to avoid frequent replacements. Technological advances in leads/electrodes have reduced energy requirements by two orders of magnitude. Micro-electronics advances sharply reduce internal current drain concurrently decreasing size and increasing functionality, reliability, and longevity. It is reported that about 600,000 pacemakers are implanted each year worldwide and the total number of people with various types of implanted pacemaker has already crossed 3 million. A cardiac pacemaker uses half of its battery power for cardiac stimulation and the other half for housekeeping tasks such as monitoring and data logging. The first implanted cardiac pacemaker used nickel-cadmium rechargeable battery, later on zinc-mercury battery was developed and used which lasted for over 2 years. Lithium iodine battery invented and used by Wilson Greatbatch and his team in 1972 made the real impact to implantable cardiac pacemakers. This battery lasts for about 10 years and even today is the power source for many manufacturers of cardiac pacemakers. This paper briefly reviews various developments of battery technologies since the inception of cardiac pacemaker and presents the alternative to lithium iodine battery for the near future.

## Introduction

### Cardiac Pacemaker

The pacemaker unit delivers an electrical pulse with the proper intensity to the proper location to stimulate the heart at a desired rate. The cardiac pacemaker comprises of a pulse generator and a lead system. The pulse generator houses electrical components responsible for generating the pulse (via output circuits) at the proper time (via timing and control circuits) based on events sensed (via sensing circuits). It also contains a power supply (battery) and may include other elements such as telemetry for testability and programmability and memory (ROM or RAM) to store data for diagnostic purposes [1].

Impulses are transmitted to the heart by means of a lead, which is attached to the pulse generator via the connector block. A lead is either unipolar or bipolar; a unipolar lead contains one insulated coil, whereas a bipolar lead contains two coils, separated by an inner insulation. An outer insulation shields a lead from the environment. The tip of a lead, which contains an electrode, is implanted into the inner, endocardial surface of the heart, the actual location depends on the type of pacemaker. The pacemaker unit  is usually implanted in the pectoral region, with the lead running through the right subclavian vein to the internal surface of the heart. A pacemaker is programmed by means of a programmer, a computer with a special user interface for data entry and display, and with special software to communicate with the pacemaker. The telemetry head is placed above the location of the pacemaker; information from the programmer to the pacemaker, and back, is transmitted by means of telemetry.

The casing of the pulse generator functions as housing for the battery and all other electronic and electrical circuits. A connector block, made of polyurethane, (glass materials were used to comprise the connector block in earlier models) is located at the top of the pacemaker. It serves to attach the pacemaker to the pacemaker lead(s). The present day pulse generator case is made of titanium, a metal that is ten times as strong as steel, but much lighter. Titanium and two of its alloys, niobium and tantalum, are biocompatible, they exhibit physical and mechanical properties superior to many other metals. The modulus of elasticity (measure of stiffness) of titanium and its alloys range between 100-120GPa. Extreme resistance to corrosion and durability make titanium and its alloys ideal materials for hermetically sealed pulse generator cases for cardiac pacemakers.

Titanium replaced ceramics and epoxy resin with silicone rubber, which were used for encapsulation of some pacemakers in the past. To assemble the pulse generator, the hybrid circuits and the battery are placed in the titanium case (ASTM Grade 1) in a specially designed clean room that has no static charge (less than 1% moisture) and no dust in it. Once the hybrid circuits and the battery are in the casing, the casing is welded shut with a high-powered laser beam. This laser beam gives the pulse generator a hermetic seal, which means that the device is airtight and liquid-tight. After welding, the top, or header of the pacemaker is attached and the entire device is covered in a thin layer of plastic (epoxy plastic). This plastic coating further seals the pacemaker.

**Figure 1:**
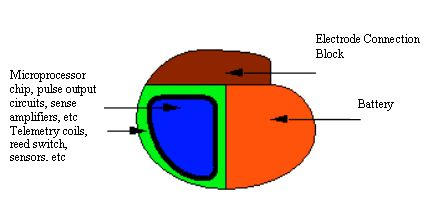
Typical Pacemaker Diagram

The casing is a given a kind of elliptical shape and a typical pacemaker diagram is shown in Figure 1.   This upgrade to titanium allowed patients to safely use appliances such as microwave ovens because titanium helps to shield the internal components and reduce the external electromagnetic interference. In addition, titanium casing shields from ground level cosmic radiation.

### Batteries for Cardiac Pacemakers

In 1958, Ake Senning, a thoracic surgeon at the Karolinska Hospital in Stockholm, implanted myocardial electrodes and a pulse generator with a rechargeable nickel-cadmium battery in a 40-year-old patient. Senning and his associate, Rune Elmquist, an engineer with the Swedish firm Elema Schonander, had developed and tested this pacemaker between 1956 and 1958 [2]. The pulse generator failed within a few hours; a successor lasted about 6 weeks. The history of the implantable cardiac pacemaker is traced from its inception in 1951, through its development and trials in 1958, to its successful implantation in 10 patients in 1960, and on to its commercial realization [3]. The usage of implanted pacemakers has been ever increasing since then. The battery occupies major portion of the pulse generator in terms of weight, volume, and size. The most important factor for a cardiac pacemaker battery is its reliability. Unlike many consumer products, batteries in implantable devices cannot be replaced. They are hard wired at the time of manufacture before the device is hermetically sealed. From that point on, the battery is expected to power the device during final testing at the factory, during the shelf life and throughout the useful life of the device while it is implanted. In general the power source of the implantable device is the only component, which has a known predictable service life, which in turn determines the service life of the implanted device itself.

It is indeed fascinating to see the breadth and the vision of the early investigators of implantable power sources [1] in the almost desperate search for a power source that would enable the pacemaker to last as long as the expected lifetime of the average patient. This paper presents a brief history and review of various types of batteries used in cardiac pacemakers since beginning. The smooth transition from zinc-mercury, nuclear batteries to the lithium-iodine batteries are presented along with product information obtained from the manufacturers. The technical advantages of lithium iodine battery in terms of its longevity, no gas generation, adaptable shapes and sizes, corrosion resistance, minimum weight, excellent current drain characteristics suitable to cardiac pacemakers are highlighted in this paper. The future of cardiac pacemaker batteries in terms of alternatives to lithium iodine battery is also presented.

### Electrochemical Power sources 

We need to generate electrical energy from some other source of energy. Chemical energy is the most practical source and is generally used in one of two possible ways. Fuels can be burnt in a heat engine or fuel cells can be used. Fuel cells have no moving parts and do not require the mechanical energy to generate Electrical energy. Chemical energy can also be stored in two types of electrochemical power sources, primary cells or batteries, and secondary cells or batteries. Primary cells are those used once and then discarded, whereas secondary cells can be discharged and recharged many times. In theory, many electrochemical reactions are reversible. In practice, only a few systems are worthwhile and safe. In general, electrochemical power sources have developed in an evolutionary manner.

### Battery Performance Parameters

The definitions for some of the important parts of a battery and its performance parameters like voltage, duty cycle, temperature, shelf life, service life, safety and reliability, internal resistance, specific energy (watt-hours/kg), specific power (watts/kg), etc are well known [4]. A good battery design is a compromise between various performance parameters to meet the requirements of the specific application. Critical factors in selecting a cardiac pacemaker battery technology are: minimum and maximum voltage, initial, average, and maximum discharge current, continuous or intermittent operation (size and duration of current pulses), long shelf and service life, high specific energy and specific power, impact, and good performance in a variety of conditions (temperatures, duty cycles, etc.). Cardiac pacemaker battery design poses special challenges in development of biocompatible materials, corrosion and sealing, light weight and flat type, high reliability, accurate end of life battery predictions, etc.

### Early Developments

Rechargeable (secondary batteries) nickel-cadmium batteries were used in the beginning (in 1958) of pacemaker implants in human beings. They were inductively recharged by the transmission of energy to the implanted receiver. The cell voltage was 1.25 V and the capacity was 190 mAh. The major problems were two fold, the first being very short life time and the second was to place the responsibility for recharging in the hands of patients, which is not a good medical practice. It was well known that primary or non-rechargeable batteries would give longer lifetime compared to secondary batteries. There are still some rechargeable pacemakers in use though not sold any more.

Some of the early pulse generators constructed mainly from discrete components were powered by series-wired mercury-zinc batteries [5]. Three to six cells in series provided 4-8 V. They were widely used at that time (around 1960s). Such mercury-zinc batteries were cast in epoxy, which was porous to the discharge of the battery released hydrogen  and permitted its dissipation, which required venting and hence could not be hermetically sealed. This allowed fluid leakage into the pacemaker at times that caused electrical shorting and premature failure. The terminal voltage decay characteristic of the mercury-zinc battery is such that normal battery depletion results in little change in the terminal voltage until the end of battery’s useful life. This makes failure difficult to anticipate [6].   This battery was improved in its design and still the life was only about two years with an abrupt drop in voltage as they become depleted. No device of this type is currently in use [7].

*Biological batteries* (which use power from within the human body) were experimented unsuccessfully1 for practical use in pacemakers.

*Nuclear batteries* were tried successfully for some period. Practical nuclear batteries use plutonium (^238^Pu). It has a half-life of 87 years so the output degrades only by 11% in 10 years [1,7]. However it is highly toxic and 1μg in the blood stream could be fatal. Early pacemakers used metallic plutonium where as later ones used ceramic plutonium oxide. The plutonium emits alpha particles, which impact upon the container and generate heat. Thermopiles of dissimilar p- or n-doped bismuth telluride generate the electricity for the pacemaker circuits. Though these nuclear power sources had very long life, they were large and created problems when travelling between states and countries due to the presence of their radioactive fuel. They also must be removed at the time of death and returned for proper disposal. Nuclear powered pacemakers are no longer sold [7] but still a small number of implanted nuclear devices that remain in use. Nuclear power sources became obsolete with the development of lithium batteries.

## Lithium Batteries

Lithium has the highest specific energy of all but it has only become possible since mid 1970s to manufacture practical batteries. Because lithium reacts violently with water, non-aqueous electrolytes must be used. Organic solvents such as acetonitrile and propylene carbonate, plus inorganic solvents such as thionyl chloride (SOCl2) are typical, with a compatible solute to provide conductivity. Many different materials such as sulfur di, thionyl chloride, manganese dioxide, and carbon monofluoride, are used for the active cathode material.

### Introduction

Introduction of a lithium iodine battery in 1975 greatly extended the pacemaker battery life (more than 10 years for some models) and replaced the mercury-zinc battery. Lithium Primary batteries are used in pacemakers since they meet the requirements of long life, low drain current and voltage characteristics. The shelf life of primary lithium cells is typically equivalent to a 10% loss of capacity over five years [1]. This compares with a similar loss for alkaline cells over only one year. The long shelf life of lithium batteries arises from the lithium metal surface becoming passivated by reaction with the electrolyte. All lithium systems are said to be thermodynamically unstable but kinetically stable. They produce no gas and hence they can be hermetically sealed. In addition, the terminal voltage decay characteristic is well behaved, falling slowly enough for battery end-of-life(EOL) to be anticipated in routine follow up.

Lithium batteries are categorized under liquid cathode cells, solid cathode cells, and solid electrolyte cells.

The *liquid cathode* systems, Li/SO2, Li/SOCl2 and Li/SO2Cl2, plus their derivatives, are capable of higher discharge rates than the solid cathode systems such as Li/MnO2 and Li/CFX. These are not suitable for applications in implanted cardiac pacemakers. However lithium sulfur dioxide batteries are used in automated external defibrillators (“AEDs”) that can restore a normal cardiac rhythm to victims of sudden cardiac arrest. *Solid Cathode* Lithium Cells use solid cathode materials such as MnO2, CuO, V2O5 and carbon monofluoride, (CF)n. They have the advantage of not being pressurized, although they cannot be discharged as rapidly as liquid cathode cells. They are available in button and cylindrical forms. About 80% (by number) of all lithium batteries in use are of the Li/MnO2 type. The energy density is similar to that of the Li/SO2 cells when discharged slowly and their slow self-discharge characteristic make them suitable for memory backup, watches, calculators, cameras, mines and munitions, etc. Voltage delay appears to be less of a problem with solid cathode cells.

The solid cathode cells do not support currents as high as the liquid cathode ones. This is because the liquid cathode undergoes a discharge at the surface of the electrode (which comprises a high surface area carbon supported on a metal mesh) where the discharge products are deposited. In contrast, discharging at a solid cathode involves diffusion of lithium ions into the bulk of the cathode, which is a slower process.

Continuous operation of liquid and solid cathode cells above 2A will lead to a significant rise in cell temperature, so this needs to be borne in mind for a particular battery application, the temperature rise being of more importance for the high pressure Li/SO2 cells. Possible hazards, like explosions associated with lithium liquid and solid cathode batteries are still a concern for absolute safety and lot of research is still going on to stipulate the rules and regulations as to how they must be disposed off towards the end of their life.

*Solid electrolyte lithium cells:* Several solids, such as lithium iodide, are electronic insulators but reasonably good ionic conductors and can be used as the electrolyte in solid electrolyte batteries. Such batteries are characterized by extremely long service life at low drain currents, even at high temperatures. They are very much suitable for applications such as cardiac pacemakers, and for preserving volatile computer memory.

Since 1972, a variety of lithium batteries have been used. These include Li/SOCI2, Lithium-silver chromate cell [Li/Ag2CrO4], lithium copper-sulfide cell[ Li/CuS], lithium – thionyl chloride cell,  Li/I2-Polyvinylpyridine (PVP), and, in more limited use, Li/LiI(Al2)3/PbI2,PbS, Pb.  In addition to their widespread use in consumer products, lithium primary batteries are the power source of choice for a range of medical implants.

 The lithium iodine-polyvinylpyride (PVP) is the principal cardiac pacemaker battery that has been in long use. The internal impedance (The resistance of a cell to an alternating current of a particular frequency) of the lithium iodine cell is an important factor in battery performance. The greater the impedance, the more difficult it is to pass current through the cell. Increased cell impedance corresponds to a decreased power source at the cell terminals. The beginning-of-life (BOL) impedance ranges from 50 to 100 Ohms. The impedance increases during service to values from 20,000 to 30,000 Ohms during the accumulation of discharge product [8].

### Lithium Iodine Battery for Cardiac Pacemaker

The lithium / iodine-polyvinylpyridine battery, first implanted in 1972 has become the power source of choice for cardiac pacemaker. Since then, improvements in cell chemistry, cell design, and modeling of cell performance have been made [10]. Cells today exhibit an energy density over three to four times as great as cells produced in 1972. Well over 3 million pacemakers have been implanted with this chemistry, and the system has exhibited excellent reliability.  The battery chemistry provides a long shelf life and high energy density. Lithium cupric sulfide was used in some pacemakers [2] manufactured by the Cordis Corporation due to its excellent energy density. However, due to the corrosive nature of this compound many abrupt pacemaker failures occurred when the battery chemicals ate through their containment. It is still present in some of the already implanted pacemakers but lithium cupric sulfide is no longer used.

Lithium Iodine has two characteristics that make it an excellent power source for cardiac pacemaker applications. The self-discharge rate is very low resulting in a long shelf life. It has a stable voltage through much of the useful life then tapers down in a gradual and predictable manner. This makes predicting the elective replacement time safe and easy.

The cathode is a complex of iodine and poly-2-vinyl pyridine (P2VP). Neither conducts electricity, but when mixed and heated at 149°C for 3 days, they react into a black viscous paste that conducts electricity. This is poured into the battery when molten and cools to form a solid. When this paste contacts metallic lithium, a monomolecular layer of crystalline lithium iodine forms. It is a molecular semiconductor that passes lithium ions, as required for current flow, but not iodine molecules [1]

### Chemical Reactions

Conventional current flows through a device from anode to cathode. For a battery, the current flows from the negative anode, through the battery, to the positive cathode. Oxidation of metal occurs at the anode, 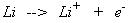  and  reduction of halide occurs at the cathode,  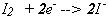. The combined reaction is, 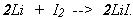 Conventional current flows from anode to cathode. The lithium reacts with iodine to form lithium-iodide, which grows in volume and increases the resistance.

### Internal Resistance

**Figure 2:**
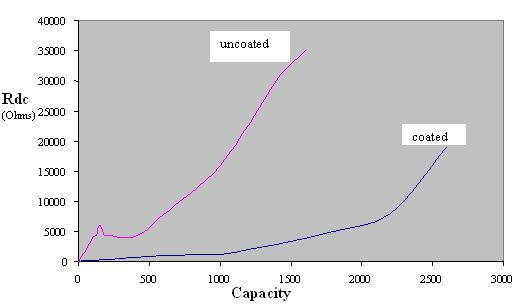
Capacity vs Internal resistance

**Figure 3:**
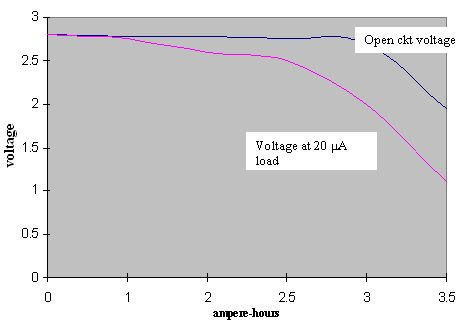
Run down characteristics

The internal cell resistance (Rdc, The resistance to flow of an electric current within a cell; the sum of the ionic and electronic resistance of the cell components) as a function of capacity for PVP- coated and uncoated (see below) lithium anode are shown in Figure 2. The open circuit voltage (OCV) and voltage at 20 μA load characteristics are shown in Figure 3. It is seen that the voltage above 2.2V (required minimum by the pacemaker electronics) is well maintained until the 2.5 Ah rating of the battery.

### Manufacturing

Lithium is easily formed into sheets that can be cut to the required sizes. It is easily pressed into specific anode shapes. The lithium anode is coated three times with a solution of PVP. The solvent is evaporated to leave a contiguous film of pure PVP on the anode surface. The precoated central lithium anode is corrugated to increase its area and lower battery impedance. To obtain lower impedance, newer designs use more concentrated active materials and larger anode surface areas. Multiple anode surfaces may be used to lower the impedance. The complex of iodine and poly-2-vinyl pyridine (P2VP) is poured into the cathode case and allowed to cool [11].

### Testing

To maintain high reliability (of the order of 0.005 % failures per month), cells are designed conservatively. They are manufactured under stringent quality controls, as demanded by the Good Manufacturing Practices (GMP) issued by the Food and Drug Administration (FDA), USA. The qualification testing is performed under accelerated test conditions specified for Li/I2-PVP cells [11]. The list includes Non-destructive examinations, thermal cycling, high pressure, mechanical vibration, temperature / humidity, mechanical shock, voltage / temperature, seal terminal strength, elevated temperature discharge, destructive analysis, and solvent resistance.

### Longevity and Battery life estimation

#### Longevity

The pacemaker battery provides energy required for the operation of the circuitry of a pacemaker, which includes the control, sensing and pulse-generating units. A major concern in using battery is its longevity. Longevity of a battery can be determined knowing battery capacity (Ah) and current drain (microamperes). The current drain is dependent on the type of electrode as well as the circuitry and type of pulse generation of the pacemaker.

#### Life Estimation

Since the longevity of a cardiac pacemaker means its battery life, it is essential to have the circuitry to identify the remaining useful life of battery in a simple and reliable manner. Monitoring of internal resistance is a convenient tool for estimation of discharge level and for predicting the approaching end-of-service.

In many pacemaker systems, circuits are provided to measure the internal resistance of the battery to deduce the remaining life. With this circuit1, the pacemaker is first switched to “test mode“ and a resistive load is applied to the battery to measure the voltage drop. The status of the battery is indicated by generating a series of test pulses. Depending on the internal voltage drop, and thus the internal resistance, the frequency of the stimulation pulse is changed, which is measured externally. However, this circuit can only be used for batteries with increasing internal resistance as the battery discharges.

To overcome the limitations of the above technique and to measure the life expectancy of a battery with constant internal resistance, another technique was proposed [1]. The  battery test circuit is provided with a pulse counter and input logic to measure the consumed charge from the operating parameters of the pacemaker and the number of pulses delivered over a period of time. During each test, the charge delivered since the last battery test is calculated based on the count in the pulse counter, which is then summed to the contents of the charge counter in memory. The content of the charge counter is a measure of the total charge consumed and provides information about the remaining life of the battery [12].

The-circuit is implemented internally in the pacemaker unit and a means is provided to report the value of the charge counter when interrogated by telemetric methods. The advantage of this method is that there is no need to alter the frequency of stimulation pulses while testing the battery.

In some models-of pacemakers, approximately twice per day the device evaluates the battery status, which is reported during follow-up; where as in some other models, battery status is automatically evaluated every 11 hours. Battery status may be displayed in the form of a gauge (showing BOL, ERT, and EOL) and longevity remaining (> 5 years to < 0.5 years in 0.5 year increments) at 100% pacing [13].

### Specifications

The battery should meet the pacemaker pulse requirements in the range of 25 μ J, a very small power (compared to 15-40 J for Implantable Cardioverter Defibrillators). The following are broad specifications.

- Open Circuit Voltage: 2.8 Volt
- Control Circuit minimal voltage: 2.2 Volt
- Control Circuit current drain: 10 μA
- EOL battery resistance: 10 k Ohms
- C_*hold*_: 10 μF
- Oscillator frequency: 167 Hz
- Duty Cycle; 16.7 %
- Ah rating: 2 Ah (typical rating)
- Reliability: 99.6% probability of survival beyond 8 years
- Failure Rate: 0.005 % failures/month

### Weight, Volume, Shape and Size

#### Weight

Half of the occupied space is consumed by the internal battery14 in cardiac pacemaker. Therefore the energy density (energy/volume) and specific energy (energy/mass) are important considerations for implantable batteries. Compared with lead, the same volume of lithium provides eight times as much electricity, at one-thirtieth the weight. The weight of a lithium-iodine battery varies from about 12.5 grams to 15.5 grams for different manufacturers [5,13,15,16] of the pacemaker unit. The variation in weight is primarily due to the longevity and current drain capabilities of the battery.

#### Volume

The volume occupied by the battery in a pacemaker (pulse generator unit) is also about half the total volume. This varies from 5 to 8 cc for the units manufactured by different manufacturers [5,13,15,16].

#### Shape

Most of the cardiac pacemakers are shaped as variations on circular or elliptical objects1 to avoid having sharp corners that might penetrate the skin or damage surrounding tissues. Therefore, the batteries in these devices are shaped to conform to the overall device geometry, and often approximate a semicircle with a radius of about 3cm and a depth of 6 to 8mm.

#### Size

Typical dimensions of an implantable cardiac pacemaker are in the range of  49 mm x 46 mm x 6 mm / 47 mm x 41 mm x 7 mm / 45 mm x 52 mm x 7 mm / 44 mm x 42 mm x 8 mm / 41 mm x 50 mm x7 mm [5,13,15,16]. The dimensions vary from one model to an another as well as from one manufacturer to an another. The battery occupies about half of the size, and volume given in the table. Most of the companies use the lithium iodine battery developed first by Wilson Great Batch [17].

## Future Batteries

Newer designs are aimed at lowering impedance by using more concentrated active materials and increasing anode surface area9. Any increase in service life of implantable medical devices, including cardiac pacemakers is highly desirable and important. In this connection it seemed worthwhile to use power sources with higher energy densities and lower internal resistance. Indeed, batteries based on other lithium systems were also proposed; lithium-silver chromate, lithium-cupric sulfide, lithium-thionyl chloride being among them. However all these batteries were rejected.

With several features being added to the implantable cardiac pacemakers and other implanted medical devices, manufacturers are going to need to pull more energy out of the battery more quickly. Today's pacemakers typically use lithium iodine batteries and defibrillators employ lithium silver vanadium oxide, next-generation systems may slowly migrate toward a newer type of lithium battery: lithium carbon monofluoride (CFx). CFx batteries reportedly offer higher energy density and can be pulsed at currents above 20 mA, which is slightly better than today's competing batteries [18] [19]. Such innovations will be necessary, particularly if OEM visions of patient management come to fruition. Medtronic, for example, has already embarked on a decade-long program, known as Vision 2010, which calls for far-reaching use of device connectivity. Ultimately, engineers say they can foresee a day when an implanted heart monitor will detect a problem and call an ambulance; all while the patient lies sleeping.

##  Lithium / carbon monofluoride (Li / CFx): a new pacemaker battery

The reduction in pacemaker size coupled with addition of more current demanding functions have motivated the development of batteries that can supply higher current densities at useful voltages than lithium / iodine batteries in use today while retaining the volumetric energy density of that system. The battery can deliver currents in the milliampere range without significant voltage drop. The system is compatible with titanium casing, allowing a 50% reduction in weight over the same size lithium / iodine battery.  Cells have been designed and tested in these laboratories and have been shown to be suitable for advanced pacemaker applications [20].

##  Lithium-polycarbon fluoride battery

This type of battery possesses very high energy density and is capable to ensure pulse discharge current as high as tens of milliamps. At the same time, in contradiction to lithium-iodine batteries, lithium-polycarbon fluoride ones use a liquid electrolyte, specifically 1 M LiBF4 in gamma- butyrolactone. This fact warrants special attention to a problem of sealing batteries for liquids and gases (due to electrolyte impurities). It is very important to check the battery leak-tightness, (meeting the reliability standards laid down for implantable cardiac pacemakers) which would qualify for medical applications. The only volatile component of lithium-polycarbon fluoride battery is gamma-butyrolactone. For detecting volatile substances, gas chromatography is viewed as the most suitable and most accurate technique. Sadly enough, gas chromatography is not able to be used for gamma-butyrolactone detection because it decomposes at a temperature below its boiling point. However, liquid chromatography is suitable for this analysis, but it is a much more sophisticated and expensive technique. Of interest, is a simple method of gamma-butyrolactone detection that was developed [21].
